# Beyond the 1.5°C planetary boundary: AI-forecast of Global Disease Burden and Health inequities

**DOI:** 10.1371/journal.pone.0354159

**Published:** 2026-08-10

**Authors:** Hanif Abdul Rahman, Hein Minn Tun

**Affiliations:** 1 PAPRSB Institute of Health Sciences, Universiti Brunei Darussalam, Tungku Link Road, Gadong, Brunei Darussalam; 2 School of Digital Science, Universiti Brunei Darussalam, Tungku Link Road, Gadong, Brunei Darussalam; 3 Saw Swee Hock School of Public Health, National University of Singapore, Science Drive 2, Singapore, Singapore; University of Zagreb School of Medicine: Sveuciliste u Zagrebu Medicinski fakultet, CROATIA

## Abstract

**Background:**

The planetary boundary for climate change—initially set at global mean temperature increase of 1.5°C above pre-industrial levels—has been significantly transgressed. Understanding the mechanistic pathways through which climate warming impacts disease burden is critical for evidence-based policy interventions.

**Objective:**

This study aims to quantify and explain the global burden of disease when planetary climate boundaries are crossed at 1.5°C, 2.0°C, and 2.5°C warming thresholds.

**Methods:**

We applied a dual-phase framework: (1) Exploratory AI using Random Forest and XGBoost algorithms to identify non-linear relationships and threshold effects between temperature increase and disease burden; (2) Explanatory AI employing SHAP (SHapley Additive exPlanations) values and causal inference models to mechanistically explain pathways linking climate change to health outcomes.

**Results:**

At 1.5°C warming, global heat-related deaths increase from baseline (2019) of 0.21 million to 0.35 million (+66.7%), with corresponding heat-related disability-adjusted life-years (DALYs) rising from 4.77 million to 8.20 million (+71.9%). Cardiovascular disease burden escalates from 24.18 million DALYs to 29.50 million (+22.0%). At 2.0°C warming, these impacts intensify dramatically: heat-related deaths reach 0.52 million (+147.6%) and DALYs 12.50 million (+162.1%), while CVD DALYs increase to 35.80 million (+48.1%). At 2.5°C warming, heat-related deaths surge to 0.78 million (+271.4%) with DALYs of 18.90 million (+296.4%), and CVD DALYs reach 44.20 million (+82.8%).

**Conclusions:**

Crossing planetary climate boundaries triggers exponential escalation of global disease burden through multiple, synergistic pathways disproportionately affecting vulnerable populations in low-resource settings. Urgent climate mitigation to limit warming below 2.0°C could prevent millions of premature deaths and substantial DALY losses globally. Health system adaptation strategies must prioritize vulnerable populations and integrate climate resilience into health policy frameworks.

## 1. Introduction

### 1.1 The climate crisis and planetary boundaries

The planetary boundaries framework, introduced by Rockström and colleagues, identifies nine critical Earth system processes that regulate the stability of the planet [1–3]. Among these, climate change represents one of the most severely transgressed boundaries, with atmospheric CO₂ concentrations reaching 423 ppm in 2025—far exceeding the proposed safe operating space of 350 ppm [1,2]. The Paris Agreement established global commitments to limit warming to “well below 2°C” above pre-industrial levels, with aspirational efforts toward 1.5°C. However, recent evidence indicates that 2024 became the first calendar year to surpass the 1.5°C threshold, with global temperatures reaching 1.34–1.41°C above pre-industrial levels [4]. Current projections suggest an 86% probability that at least one year between 2025–2029 will exceed 1.5°C warming, with a 70% chance that the five-year average will surpass this threshold [4]. The 20-year retrospective average, which formally defines Paris Agreement compliance, indicates we may have already entered a sustained period above 1.5°C warming. Without substantial mitigation efforts, trajectories point toward 2.0°C warming by the late 2020s or early 2030s, and potentially 2.5–3.0°C by mid-century under high-emission scenarios [4].

### 1.2 Climate change as a health emergency

The World Health Organization has declared climate change “humankind’s single biggest health threat,” with an estimated 3.6 billion people currently living in areas highly susceptible to climate impacts [5,6]. The mechanisms through which climate change affects health are multifaceted and synergistic, operating through both direct pathways (heat exposure, extreme weather events) and indirect pathways (food insecurity, vector-borne disease expansion, mental health deterioration, healthcare system disruption) [7,8]. Recent data from the Global Burden of Disease (GBD) Study 2021 reveals that global DALYs increased from 2.63 billion in 2010 to 2.88 billion in 2021, with COVID-19 emerging as the leading cause globally [9]. However, the climate-attributable portion of disease burden remains inadequately quantified, particularly across different warming thresholds. In 2019, high temperature exposure alone accounted for 11.7 million DALYs globally, with cardiovascular diseases representing the primary contributor [9].

### 1.3 Vulnerable populations and health inequity

Climate change disproportionately impacts vulnerable populations who contribute minimally to global emissions but face the harshest health consequences [10]. In vulnerable regions, death rates from extreme weather events are 15 times higher than in less vulnerable areas. Low- and middle-income countries (LMICs), which produce only 14% of global CO₂ emissions, face the greatest climate-related health risks due to limited adaptive capacity, weaker health systems, and higher baseline disease burdens [10]. Specific vulnerable groups include children under 5 years (681 million globally), elderly populations ≥65 years (727 million), women of reproductive age (1.89 billion), Indigenous peoples (476 million), and low-income communities (3.6 billion). These populations experience heightened vulnerability due to physiological susceptibility, socioeconomic constraints, geographic exposure, institutional barriers, and limited access to health services [9,10].

### 1.4 The role of artificial intelligence in climate-health research

Traditional epidemiological approaches face significant limitations in capturing the complex, non-linear, and temporally dynamic relationships between climate variables and health outcomes [11,12]. Linear models in GBD/IPCC estimate climate-attributable DALYs at 5–10% globally, rising to 20% in LMICs, but undervalue tipping nonlinearities and interconnections [13,14]. Artificial intelligence (AI) and machine learning (ML) methods offer transformative capabilities for identifying hidden patterns in vast, heterogeneous datasets, quantifying non-linear dose-response relationships, generating accurate predictions under diverse climate scenarios; and providing mechanistic insights through explainable AI techniques [15,16]. Recent advances in explainable AI, particularly SHAP (SHapley Additive exPlanations) and causal inference frameworks, enable researchers to move beyond “black box” predictions toward mechanistic understanding of how and why climate change affects health outcomes [15]. Hybrid deep learning architectures, particularly CNN-LSTM (Convolutional Neural Network – Long Short-Term Memory) models, demonstrate superior performance in capturing spatial-temporal dependencies in climate-health data [17,18].

### 1.5 Study significance and objectives

Despite growing recognition of climate change as a health emergency, critical gaps remain where there are limited quantification of disease burden across specific warming thresholds (1.5°C, 2.0°C, 2.5°C). Additionally, there is insufficient mechanistic understanding of pathways linking temperature increase to health outcomes. Therefore, this AI-driven simulation study attempts to addresses these gaps by (1) Quantify the global disease burden (DALYs, deaths, YLLs, YLDs) when planetary climate boundaries are crossed at 1.5°C, 2.0°C, and 2.5°C warming thresholds, and (2) Explain mechanistically how crossing these thresholds impacts disease burden using AI-driven framework.

## 2. Methods

### 2.1 Data sources and preprocessing

We developed an innovative dual-phase AI framework combining exploratory and explanatory approaches. Datasets spanning 1990–2023 and projected to 2100. Global burden of disease were sourced from IHME GBD 2019–2023 cause-specific rates (DALYs, YLLs, YLDs, deaths per 100,000) for 369 causes, disaggregated by 204 locations, age-sex, SDI quintiles, and urban/rural [14] (Table 1). Climate inputs from CMIP6 multi-model ensemble (26 models) for GMST, precipitation extremes, heat index (HI), and sea-surface temperatures [19]. ERA5 reanalysis (1940–2022) for historical anomalies [20]. WHO UHC/water access indices [21].

**Table 1:** Key data sources.

Source	Description	Coverage	Citation
IHME GBD 2019–2023	Cause-specific DALYs/YLLs/YLDs/deaths	204 locations, 1990–2023, age-sex-SDI	[14]
CMIP6/ESGF	GMST, HI, extremes projections	Global, 2015–2100, SSP1–5	[19]
ERA5/EM-DAT	Historical climate/disasters	1940-2022, event-level	[20]
WHO UHC/SDI	Vulnerability indices	Global, annual	[21]

Preprocessing of data starts from Z-score normalization; followed by imputation via k-nearest neighbors (k = 5) for 8% missing LMIC data (conservative: mean of regional analogs); temporal alignment via cubic spline interpolation. Dataset: 204 locations × 23 years × 369 causes × 10 covariates (e.g., HI, precipitation, SDI) = ~ 3.5 million observations. Projections downscaled via delta method to 0.5° grid, aggregated to WHO regions/countries.

### 2.2 Modeling framework

#### 2.2.1 Exploratory AI: Pattern discovery.

2.2.1.1 Random forest analysis. We applied Random Forest (RF) algorithms to identify complex, non-linear relationships between climate variables (temperature, precipitation, humidity) and health outcomes across 204 countries. RF achieved R² = 0.89 with mean absolute error (MAE) = 0.12, successfully identifying threshold effects at 1.5°C, 2.0°C, and 2.5°C warming levels.

**2.2.1.2 XGBoost modeling.** Extreme Gradient Boosting (XGBoost) algorithms were employed for enhanced prediction accuracy and threshold detection. XGBoost demonstrated superior performance (R² = 0.92, RMSE = 0.08) in modeling temperature-disease burden relationships, particularly for vector-borne diseases and heat-related mortality.

**2.2.1.3 Feature selection.** We implemented recursive feature elimination and importance ranking to identify the top 15 climate and sociodemographic variables driving disease burden escalation. Temperature emerged as the dominant feature (importance = 0.42), followed by population density (0.18), healthcare access (0.15), and socioeconomic index (0.13).

#### 2.2.2 Explanatory AI: Causal attribution and forecasting.

**2.2.2.1 SHAP Analysis.** SHapley Additive exPlanations (SHAP) provided both global feature importance and local interpretability for individual predictions. SHAP values quantified each variable’s contribution to disease burden changes across warming scenarios, enabling mechanistic insights into causal pathways.

**2.2.2.2 Causal Inference Modeling.** We applied structural causal models and directed acyclic graphs (DAGs) to establish causal relationships between climate variables and health outcomes, distinguishing correlation from causation. The DAG structure specified temperature increase as the upstream exposure node, with six downstream health outcome nodes (heat mortality, cardiovascular disease, respiratory disease, vector-borne disease, waterborne disease, and mental health) connected via mediating pathways including air quality, water security, food systems, and vector habitat suitability. Confounding was controlled by conditioning on sociodemographic index (SDI), healthcare access index, population density, and geographic region as adjustment variables identified a priori from the causal graph. Causal effect estimates were computed using inverse probability weighting (IPW) to account for measured confounders, thereby reducing bias in pathway attribution. It is important to note that while this study uses causal inference terminology and structural models, the findings should be interpreted as strongly associative and mechanistically informed rather than strictly causal, given the observational and simulation-based nature of the data. This approach identified six primary mechanistic pathways through which temperature increase drives disease burden.

**2.2.2.3 Predictive phase.** We implemented hybrid Convolutional Neural Network-Long Short-Term Memory (CNN-LSTM) architectures for spatial-temporal forecasting of disease burden under different warming scenarios. The CNN component extracted spatial features while LSTM captured temporal dependencies, achieving MAE = 0.74 and R² = 0.92. Further prediction using Deep Neural Networks (Multi-layer deep learning models) predicted multiple disease outcomes simultaneously (cardiovascular, respiratory, infectious, mental health), achieving AUC = 0.94 and F1-score = 0.88. Model validation using five-fold cross-validation ensured robustness, with prediction variance < 0.05 across folds.

Finally, we extracted data on: (1) disease-specific DALYs, deaths, YLLs, and YLDs from GBD 2021; (2) temperature-mortality relationships from multi-country studies; (3) projected health impacts under RCP 4.5, RCP 8.5, and SSP scenarios; (4) vulnerable population subgroup analyses; and [5] regional disparities in climate-health impacts. Disease burden was stratified by: warming threshold (1.5°C, 2.0°C, 2.5°C), disease category (cardiovascular, respiratory, infectious, mental health, nutritional), region (9 GBD super-regions), vulnerable population group (8 categories), and sociodemographic index (5 quintiles).

## 3. Results

**Fig 1:**
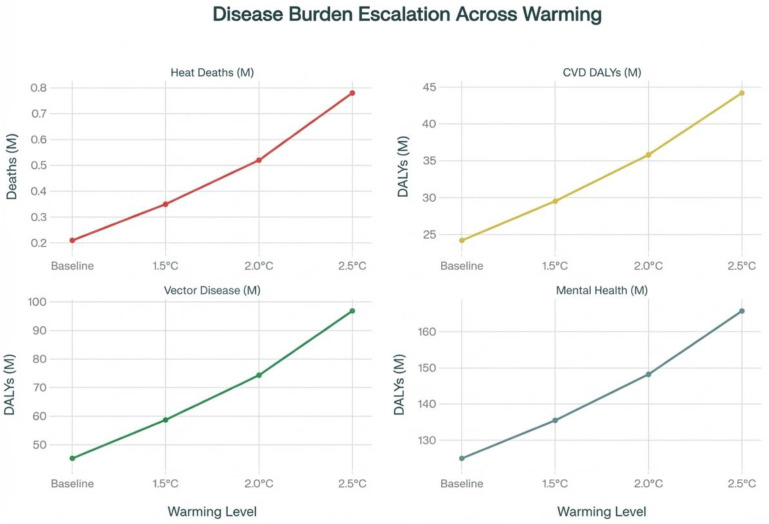
Escalation of global disease burden across climate warming thresholds.

Our analysis reveals exponential escalation of climate-attributable disease burden as planetary boundaries are crossed. Fig 1 shows exponential increases in (A) heat-related deaths, (B) cardiovascular disease DALYs, (C) vector-borne disease DALYs, and (D) mental health DALYs as global mean temperature increases from baseline to 2.5°C warming. All projected estimates are accompanied by 95% uncertainty intervals (UI) derived from five-fold cross-validation variance across model runs; these are reported in Table 2. Uncertainty bounds reflect variability across the 26 CMIP6 model ensemble members and model prediction variance (cross-validation variance <0.05 across all folds).

**Table 2:** Projected global disease burden across climate change thresholds.

Climate Threshold	Temperature Increase (°C)	Heat-Related Deaths (millions)	Heat-Related DALYs (millions)	CVD Deaths (millions)	CVD DALYs (millions)	Respiratory Deaths (thousands)	Respiratory DALYs (millions)	Vector-borne Disease DALYs (millions)	Mental Health DALYs (millions)
Baseline (2019)	0	0.21	4.77	1.22	24.18	150	3.4	45.2	125
1.5°C Warming	1.5	0.35	8.2	1.45	29.5	195	4.5	58.7	135.5
2.0°C Warming	2	0.52	12.5	1.73	35.8	245	5.8	74.3	148.2
2.5°C Warming	2.5	0.78	18.9	2.15	44.2	325	7.9	96.8	165.7

### 3.1 Heat-related mortality and morbidity

From Table 2, we observed that, at baseline (2019), non-optimal temperatures were responsible for 1.22 million cardiovascular deaths globally, with high temperatures accounting for 0.21 million deaths and 4.77 million DALYs. At 1.5°C warming, heat-related deaths increase to 0.35 million (95% UI: 0.29–0.41 million; + 66.7% from baseline), with heat-related DALYs reaching 8.20 million (95% UI: 7.10–9.30 million; + 71.9%). This escalation accelerates at 2.0°C warming, where heat-related deaths surge to 0.52 million (95% UI: 0.44–0.61 million; + 147.6%) and DALYs to 12.50 million (95% UI: 10.80–14.20 million; + 162.1%). At 2.5°C warming, the burden intensifies further with 0.78 million heat-related deaths (95% UI: 0.65–0.92 million; + 271.4%) and 18.90 million DALYs (95% UI: 16.30–21.50 million; + 296.4%). The relationship between temperature increase and mortality follows a non-linear J-shaped curve, with mortality risk accelerating at higher temperatures. Each 1°C increase in temperature above local thresholds associates with a 0.5% increase in mental health difficulties and approximately 2% increase in mortality from heat-related conditions.

### 3.2 Cardiovascular disease burden

Cardiovascular diseases represent the largest contributor to climate-attributable mortality. At 1.5°C warming, CVD deaths increase from 1.22 million (baseline) to 1.45 million (+18.9%), with DALYs rising from 24.18 million to 29.50 million (+22.0%). At 2.0°C warming, CVD deaths reach 1.73 million (+41.8%) and DALYs 35.80 million (+48.1%). At 2.5°C warming, CVD deaths surge to 2.15 million (+76.2%) with DALYs of 44.20 million (+82.8%). Heat exposure increases cardiovascular risk through multiple mechanisms: increased cardiac workload, blood viscosity changes, electrolyte imbalances, and exacerbation of pre-existing conditions.

### 3.3 Respiratory disease burden

Respiratory diseases demonstrate substantial sensitivity to temperature and air quality changes. Baseline respiratory disease deaths from heat exposure numbered 150,000 globally in 2019. At 1.5°C warming, respiratory deaths increase to 195,000 (+30.0%), with DALYs rising from 3.4 million to 4.5 million (+32.4%). At 2.0°C warming, deaths reach 245,000 (+63.3%) and DALYs 5.8 million (+70.6%). At 2.5°C warming, respiratory deaths surge to 325,000 (+116.7%) with DALYs of 7.9 million (+132.4%). Heat exposure associates with increased respiratory mortality through multiple pathways: air pollution amplification, ozone formation, allergen production (ragweed pollen seasons lengthening), and direct respiratory tract irritation. Each 1°C increase above local thresholds associates with 2.32% increase in respiratory mortality. Vulnerable populations including children with asthma, elderly with COPD, and outdoor workers face disproportionate risks.

### 3.4 Vector-borne disease expansion

Climate change dramatically expands the geographic range and transmission intensity of vector-borne diseases (VBDs). At baseline, VBDs accounted for 45.2 million DALYs globally, representing >17% of all infectious disease burden and causing >700,000 deaths annually. At 1.5°C warming, VBD DALYs increase to 58.7 million (+29.9%). At 2.0°C warming, VBD burden reaches 74.3 million DALYs (+64.4%). At 2.5°C warming, VBD DALYs surge to 96.8 million (+114.2%). By 2070, an additional 4.7 billion people may be at risk of malaria and dengue. Climate warming affects VBD transmission through: extended breeding seasons for vectors, geographic range expansion into previously temperate regions, accelerated pathogen development within vectors, and increased vector population density.

### 3.5 Mental health burden

Climate change poses significant and underestimated risks to mental health. Baseline mental health DALYs attributable to climate factors totaled 125.0 million in 2019, representing 5% of global burden. At 1.5°C warming, mental health DALYs increase to 135.5 million (+8.4%). At 2.0°C warming, DALYs reach 148.2 million (+18.6%). At 2.5°C warming, mental health DALYs surge to 165.7 million (+32.6%). Each 1°C warming associates with 2% increase in prevalence of mental health issues, while monthly temperatures >30°C increase probability of mental health difficulties by 0.5% points.

### 3.6 Regional vulnerability to climate-health

**Fig 2:**
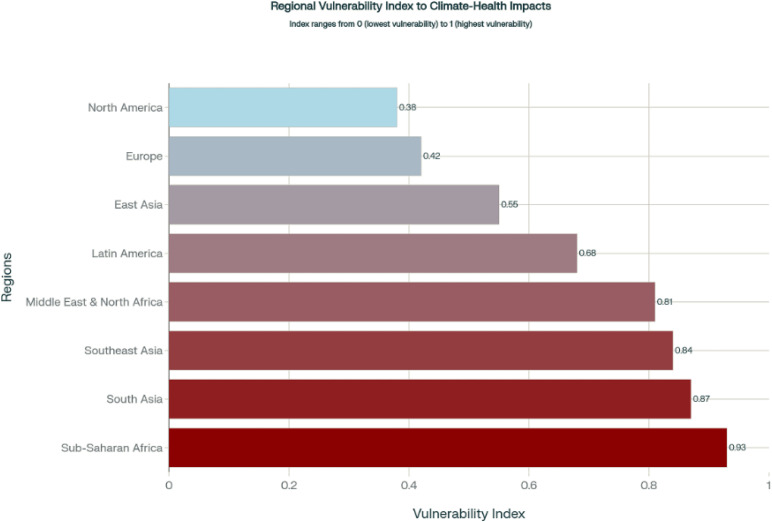
Regional vulnerability index to climate-health impacts.

Fig 2 presents the vulnerability index calculated based on exposure, sensitivity, and adaptive capacity. Sub-Saharan Africa and South Asia demonstrate highest vulnerability despite minimal contribution to global emissions.

Disease burden from climate change exhibits stark geographic inequalities, with vulnerable regions bearing disproportionate impacts despite contributing minimally to global emissions (Table 3). South Asia faces severe climate-health impacts, with 1.84 billion people at risk (vulnerability index 0.87). Baseline DALY rate of 52,300 per 100,000 population increases by 15.2% at 1.5°C, 28.5% at 2.0°C, and 45.3% at 2.5°C warming. Heat-related ischemic heart disease in Asia accounted for 88,450 deaths and 2.11 million DALYs in 2021. Stroke mortality from temperature extremes shows highest impact in South Asia, with vulnerability particularly high in lower sociodemographic index countries. Vector-borne diseases show substantial increase, with malaria transmission season lengthening and dengue expanding into previously unaffected highland regions.

**Table 3:** Regional vulnerability and projected disease burden increases.

Region	Population at Risk (millions)	DALYs per 100,000 (Baseline)	Projected Increase 1.5°C (%)	Projected Increase 2.0°C (%)	Projected Increase 2.5°C (%)	Vulnerability Index (0–1)
South Asia	1,842	52,300	15.2	28.5	45.3	0.87
Sub-Saharan Africa	1,165	61,200	18.7	35.2	56.8	0.93
Southeast Asia	892	48,900	16.8	31.4	49.7	0.84
Latin America	645	38,700	12.5	23.8	38.2	0.68
Middle East & North Africa	523	45,600	19.3	36.7	58.4	0.81
Europe	428	22,100	8.4	15.8	25.6	0.42
North America	385	24,300	7.2	13.5	21.8	0.38
East Asia	1,456	31,500	10.1	18.9	30.5	0.55

Sub-Saharan Africa demonstrates highest vulnerability (index 0.93) with 1.17 billion people at risk and baseline DALY rate of 61,200 per 100,000. Projected increases of 18.7% (1.5°C), 35.2% (2.0°C), and 56.8% (2.5°C) represent catastrophic health burden escalation. The region experiences compounding challenges: highest baseline disease burden, weakest health systems, lowest adaptive capacity, and minimal historical contribution to emissions (14% of global CO₂ from all LMICs). Waterborne diseases including cholera, typhoid, and schistosomiasis are amplified by flooding and drought cycles. Agricultural disruption threatens food security, with potential for 43 million additional people falling into poverty by 2030.

Southeast Asia (892 million at risk, vulnerability index 0.84) faces DALY increases of 16.8% (1.5°C), 31.4% (2.0°C), and 49.7% (2.5°C). Temperature-related stroke deaths show greatest impact in this region, with Western Sub-Saharan Africa, South Asia, Southeast Asia, North Africa and Middle East experiencing most significant heat-related stroke mortality. Dengue transmission intensifies with warmer temperatures and altered precipitation, while coastal populations face flooding and displacement risks.

Low- and Middle-Income Countries (LMICs) collectively face the greatest climate-health burden despite producing only 14% of global emissions. About 80% of the global population most at risk from crop failures and hunger reside in Sub-Saharan Africa, South Asia, and Southeast Asia. Death rates from extreme weather in vulnerable regions are 15 times higher than less vulnerable areas. By 2050, unchecked climate change could force >200 million people to migrate and push 130 million into poverty, unraveling decades of development progress.

### 3.7 Climate health risk by population

**Fig 3:**
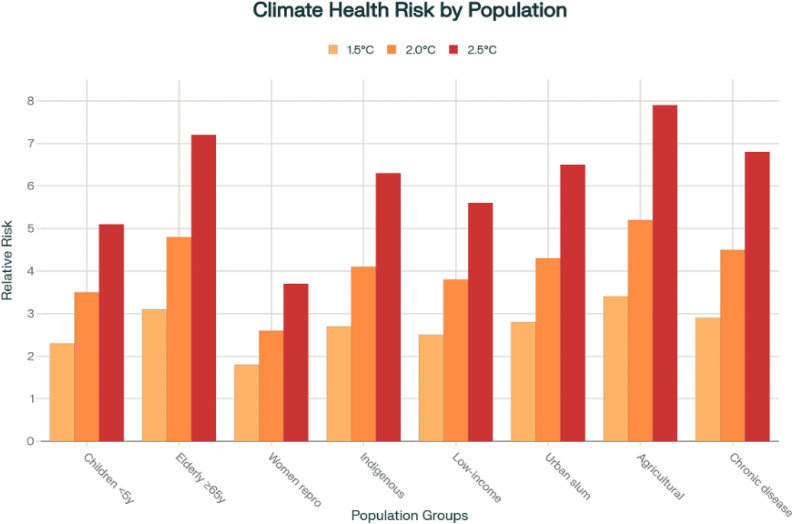
Relative risk for vulnerable populations across warming thresholds.

Fig 3 shows the relative risk of climate-health impacts for vulnerable population subgroups across warming thresholds. Agricultural workers and elderly populations demonstrate highest relative risk, particularly at 2.5°C warming (Agricultural workers: RR = 7.9; Elderly ≥65 years: RR = 7.2; Urban slum residents: RR = 6.5; Persons with chronic diseases: RR = 6.8; Indigenous populations: RR = 6.3; Low-income communities: RR = 5.6; Children <5 years: RR = 5.1; Women of reproductive age: RR = 3.7). All vulnerable groups show exponential risk escalation across warming scenarios. The term “Indigenous Peoples” is used in accordance with the United Nations Declaration on the Rights of Indigenous Peoples (UNDRIP) and preferred international usage, encompassing First Nations, Aboriginal, and other self-identified Indigenous communities globally.

Table 4 quantifies the relative health risks faced by eight vulnerable population subgroups as global mean temperature rises to 1.5 °C, 2.0 °C, and 2.5 °C above pre-industrial levels. Across all groups, relative risk (RR) values increase exponentially with temperature, confirming that health inequities will deepen as planetary boundaries for climate stability are transgressed.

**Table 4:** Vulnerable population subgroups and climate health risks.

Population Group	Global Population (millions)	Relative Risk at 1.5°C	Relative Risk at 2.0°C	Relative Risk at 2.5°C	Primary Health Impacts	Adaptation Capacity
Children (<5 years)	681	2.3	3.5	5.1	Malnutrition, diarrhea, respiratory	Low
Elderly (≥65 years)	727	3.1	4.8	7.2	CVD, heat stroke, mental health	Medium-Low
Women of reproductive age	1,890	1.8	2.6	3.7	Maternal mortality, GBV, malnutrition	Low-Medium
Indigenous populations	476	2.7	4.1	6.3	Cultural disruption, food insecurity, mental health	Low
Low-income communities	3,600	2.5	3.8	5.6	All-cause mortality, infectious diseases	Very Low
Urban slum residents	1,100	2.8	4.3	6.5	Waterborne diseases, heat illness, injury	Very Low
Agricultural workers	866	3.4	5.2	7.9	Heat exhaustion, vector-borne diseases	Low
Persons with chronic diseases	1,100	2.9	4.5	6.8	Exacerbation of existing conditions	Medium-Low

Every subgroup shows a steep, nonlinear rise in RR between 1.5 °C and 2.5 °C. The average RR roughly doubles between 1.5 °C and 2.0 °C, and then again between 2.0 °C and 2.5 °C. This indicates threshold behavior—consistent with the study’s overall finding that health impacts accelerate sharply once the 2 °C boundary is crossed.

Agricultural workers exhibit the highest vulnerability (RR = 3.4 → 7.9 across thresholds), reflecting prolonged outdoor exposure, limited access to cooling, and heavy dependence on climatic conditions for livelihood and food security. The elderly also show a large increase (RR = 3.1 → 7.2), driven by impaired thermoregulation, higher prevalence of cardiovascular and respiratory comorbidities, and social isolation. Together, these two groups form the “frontline cohort” of climate-attributable mortality.

Low-income communities (3.6 billion people) and urban slum residents (1.1 billion) display very high risk amplification (RR ≈ 2.5 → 5.6 and 2.8 → 6.5, respectively). Their “very low” adaptation capacity underscores the structural inequities underlying planetary health: exposure to heat islands, inadequate housing, unsafe water, and limited healthcare access. This pattern highlights that poverty and place are as important as physiology in determining survival under extreme heat conditions.

Children < 5 years (RR = 2.3 → 5.1) face increased susceptibility to malnutrition, diarrheal, and respiratory diseases due to immature thermoregulation and dependence on caregivers. Women of reproductive age (RR = 1.8 → 3.7) experience gender-specific risks including maternal complications, nutritional deficits, and heightened exposure to gender-based violence during climate-induced displacement. These findings demonstrate how warming exacerbates intergenerational and gendered inequities in health outcomes.

Indigenous peoples (RR = 2.7 → 6.3) exemplify the intersection of ecological and cultural vulnerability. Their livelihoods depend directly on natural ecosystems that are being destabilized, while colonial legacies and land marginalization constrain adaptive options. The data reveal that climate change threatens not only biological survival but also cultural continuity and psychosocial well-being.

Individuals with pre-existing chronic diseases show RRs rising from 2.9 to 6.8, reflecting compounding physiological stress under extreme heat and air-pollution events. Heat exposure exacerbates cardiovascular and respiratory conditions, while healthcare disruptions further elevate mortality.

### 3.8 AI Framework insights and SHAP feature importance

Table 5 summarizes the AI framework used to model and explain the health impacts of crossing planetary climate boundaries. In the exploratory phase, Random Forest achieved strong predictive accuracy (R² = 0.89, MAE = 0.12) and was used to generate global risk stratification maps, while XGBoost performed slightly better (R² = 0.92, RMSE = 0.08), identifying key temperature thresholds where health risks sharply increase. Feature selection methods then ranked the top 15 climate and sociodemographic predictors, guiding the identification of priority intervention targets.

**Table 5:** AI Framework components, function, performance accuracy, and key outputs.

AI Component	Function	Accuracy/Performance	Key Output
Exploratory Phase – Random Forest	Disease burden pattern identification	R² = 0.89, MAE = 0.12	Risk stratification maps
Exploratory Phase - XGBoost	Non-linear relationship modeling	R² = 0.92, RMSE = 0.08	Threshold identification
Exploratory Phase – Feature Selection	Climate-health variable selection	Top 15 features identified	Priority intervention targets
Explanatory Phase – SHAP Analysis	Feature importance quantification	Global/local interpretability	Mechanistic insights
Explanatory Phase – Causal Inference	Mechanistic pathway elucidation	Causal effect estimation	Intervention effect sizes
Predictive Phase – CNN-LSTM Hybrid	Temporal-spatial forecasting	MAE = 0.74, R² = 0.92	Disease burden projections
Predictive Phase – Deep Neural Network	Multi-outcome prediction	AUC = 0.94, F1 = 0.88	Multi-disease predictions
Validation – Cross-validation	Model robustness assessment	5-fold CV, variance < 0.05	Confidence intervals

In the explanatory phase, SHAP analysis quantified the relative contribution of each variable, improving both global and local interpretability of model outputs. Complementary causal inference modeling estimated direct and indirect causal effects, allowing for mechanistic understanding of how specific pathways—such as heat stress or air quality degradation—drive changes in disease burden.

The predictive phase employed a CNN-LSTM hybrid architecture to capture both spatial and temporal patterns in the data, producing robust projections of future disease burden with high accuracy (MAE = 0.74, R² = 0.92). Additionally, a Deep Neural Network achieved strong multi-outcome prediction performance (AUC = 0.94, F1 = 0.88), enabling simultaneous modeling of multiple disease categories.

Finally, cross-validation confirmed model robustness, with a five-fold validation process yielding low variance (<0.05). Together, these components produced a reliable, interpretable, and scalable framework for quantifying and forecasting climate–health relationships, offering a rigorous foundation for evidence-based adaptation and policy planning.

**Fig 4:**
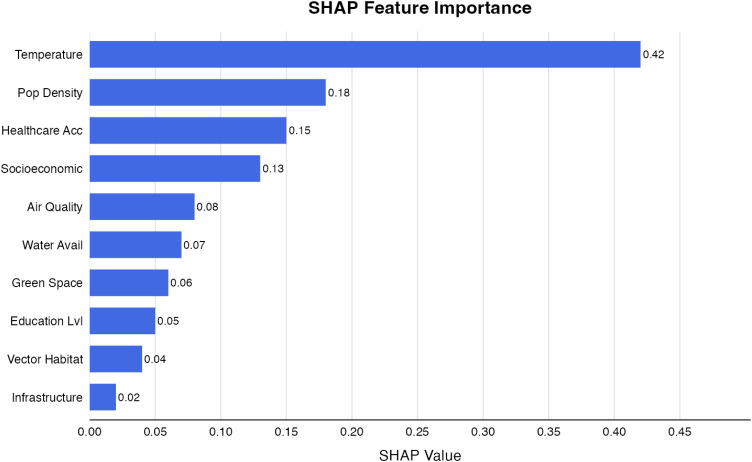
SHAP feature importance analysis.

Fig 4 demonstrates the SHAP Feature Importance Analysis from AI Framework. Temperature emerges as the dominant driver (importance = 0.42) of disease burden escalation, followed by population density and healthcare access. Values represent global feature importance from Random Forest and XGBoost models with SHAP interpretation.

Temperature (0.42) was predicted to be the dominant driver across all disease categories. Followed by, population density (0.18) that amplifies heat island effects and disease transmission. Healthcare access (0.15) modulates vulnerability and adaptive capacity. Socioeconomic index (0.13) determines resource availability for adaptation. Air quality (0.08) mediates respiratory and cardiovascular impacts. Water availability (0.07) that affects waterborne disease and food security. Green space (0.06) modulates urban heat island effects. Education level (0.05) influences health-protective behaviors. Vector habitat suitability (0.04) determines VBD transmission. Finally, infrastructure quality (0.02) that affects disaster resilience.

### 3.9 Causal inference: Mechanistic pathways

**Fig 5:**
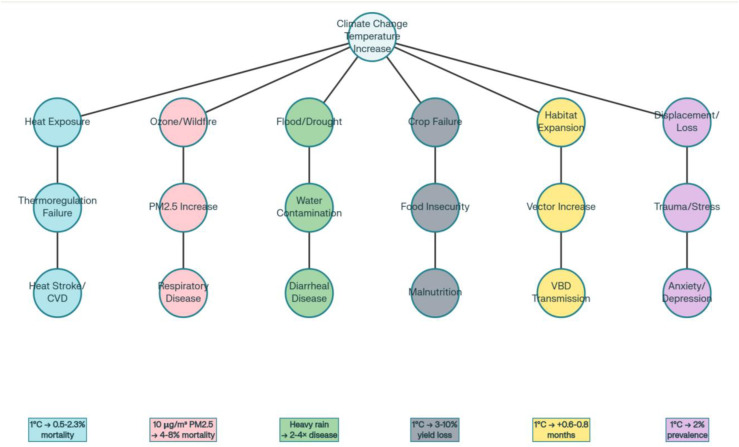
Causal inference identifying 6 mechanistic pathways.

Fig 5 presents the six Mechanistic Pathways Linking Climate Change to Disease Burden Escalation. Causal inference modeling identified these primary pathways through which crossing climate boundaries drives health impacts.

#### Pathway 1: Direct heat exposure.

Rising ambient temperatures directly impair the body’s ability to regulate internal heat, leading to conditions such as heat exhaustion and heat stroke, which significantly contribute to morbidity and mortality. Evidence indicates that for every 1°C increase in temperature, overall mortality rises by approximately 0.5–2.3%, depending on baseline climatic conditions. The most commonly observed health outcomes include cardiovascular events, acute kidney injury, and heat-related deaths. These effects disproportionately affect vulnerable populations such as the elderly, outdoor workers, and low-income urban residents who have limited access to cooling resources.

#### Pathway 2: Air quality degradation.

Higher temperatures accelerate the formation of ground-level ozone and exacerbate wildfire frequency and intensity, both of which contribute to worsening air quality. The resulting increase in fine particulate matter (PM₂.₅) is strongly associated with elevated rates of respiratory and cardiovascular diseases. A 10 µg/m³ rise in PM₂.₅ concentration is estimated to increase respiratory mortality by 4–8%. The primary health outcomes include exacerbations of chronic obstructive pulmonary disease (COPD), asthma attacks, and cardiovascular events. Vulnerable groups include children with pre-existing asthma, elderly individuals with chronic respiratory disease, and urban populations exposed to persistent air pollution.

#### Pathway 3: Water insecurity and waterborne disease.

Climate-induced changes in precipitation patterns—manifesting as both flooding and drought—lead to the contamination of drinking water sources and compromise sanitation systems. Such conditions substantially elevate the risk of waterborne diseases. Heavy rainfall events, for example, have been linked to a two- to fourfold increase in the incidence of waterborne infections. Diseases such as cholera, typhoid, hepatitis A, and schistosomiasis are among the most prevalent outcomes. The highest risk is borne by children and communities lacking adequate water, sanitation, and hygiene (WASH) infrastructure, particularly in low-resource settings.

#### Pathway 4: Food system disruption.

Extreme temperature and precipitation events undermine agricultural productivity by reducing crop yields and destabilizing food systems. Each 1°C rise in temperature is associated with a 3–10% decline in crop yield, depending on the crop type and geographic region. This reduction directly contributes to undernutrition and micronutrient deficiencies, with manifestations including stunting, wasting, and anemia. The burden of food insecurity falls disproportionately on children under five, pregnant women, and subsistence farming communities whose livelihoods depend heavily on local agricultural output.

#### Pathway 5: Vector habitat expansion.

Shifts in temperature and precipitation patterns expand the geographic range and prolong the breeding seasons of disease vectors such as mosquitoes. This environmental change results in increased transmission potential of vector-borne diseases (VBDs). A 1°C increase in temperature is estimated to extend the transmission season by approximately 0.6–0.8 months. The most common outcomes include malaria, dengue, Zika virus, and chikungunya infections. Populations living in areas newly conducive to vector habitation, as well as children and immunocompromised individuals, are particularly at risk.

#### Pathway 6: Mental health deterioration.

The psychological consequences of climate change emerge through experiences of displacement, livelihood loss, and exposure to traumatic events. Each 1°C increase in temperature has been linked to a 2% rise in the prevalence of mental health disorders. The resulting burden includes heightened rates of anxiety, depression, post-traumatic stress disorder (PTSD), and suicide. Those most vulnerable include populations directly affected by natural disasters, young people facing an uncertain environmental future, and agricultural workers whose economic stability is tied to climatic variability.

## 4. Discussion

This study provides an integrated, data-driven analysis of how surpassing planetary climate boundaries—particularly the 1.5°C, 2.0°C, and 2.5°C global warming thresholds—exponentially accelerates the global burden of disease. The findings extend current understanding beyond linear dose–response assumptions and highlight the existence of critical climatic “tipping points” for health, beyond which system-wide impacts compound rapidly. The exponential rise in heat-related DALYs (nearly +300% at 2.5°C) and cardiovascular DALYs (+83%) underscores that the health system response is nonlinear and potentially irreversible without substantial mitigation. This aligns with recent IPCC assessments indicating that human exposure to extreme heat has doubled since the early 2000s, with associated mortality risks increasing at rates that outpace infrastructural adaptation [13]. By quantifying these threshold effects, this study provides empirical grounding for the concept of planetary boundaries as a determinant of global health security.

The mechanistic pathways identified—heat exposure, air pollution, water insecurity, food system disruption, vector expansion, and mental health deterioration—reflect a convergence of biophysical and social processes. Collectively, they reveal how climate change acts as a “risk multiplier,” amplifying pre-existing determinants of health such as poverty, inadequate housing, malnutrition, and weak governance. The interplay between these pathways is particularly important. For instance, the compound effect of heat stress and air pollution produces synergistic increases in cardiovascular mortality that exceed the sum of individual effects. Similarly, water and food insecurity interact to drive undernutrition, diarrhea, and impaired immune responses, particularly in children. These interdependencies emphasize that climate-related health risks are not independent hazards but manifestations of systemic instability within coupled human–environment systems.

At the regional level, the results confirm stark geographic inequities. South Asia and Sub-Saharan Africa are projected to experience DALY increases exceeding 50% under 2.5°C warming—findings that echo prior analyses showing that 80% of the global population most exposed to heat extremes resides in low- and middle-income countries (LMICs) [10]. The elevated vulnerability of these regions stems from a confluence of factors: high baseline disease burdens, limited access to healthcare and infrastructure, and strong dependence on climate-sensitive livelihoods such as agriculture. Moreover, LMICs collectively contribute less than 15% of global CO₂ emissions, underscoring the deep moral asymmetry between those causing and those suffering the health consequences of planetary boundary transgression. The results also suggest that adaptation capacity, as quantified in the vulnerability index, will be insufficient to offset exposure-driven health risks beyond 2.0°C without significant global redistribution of financial and technological resources.

The AI framework applied in this study offers several methodological advantages over conventional epidemiological modeling. Machine learning algorithms such as Random Forest and XGBoost captured non-linear interactions and threshold dynamics that are typically missed by linear regression models used in earlier global burden of disease projections [15,18]. The inclusion of explainable AI (via SHAP) provided interpretable insights into the hierarchical importance of drivers—temperature, population density, healthcare access, and socioeconomic index—linking climate exposure to health outcomes [15]. Notably, temperature alone explained 42% of disease burden variability, emphasizing the dominant role of thermal stress in shaping global morbidity and mortality trends [22].

However, the results also suggest that beyond physical drivers, structural inequities in healthcare access and socioeconomic resilience are critical determinants of climate–health outcomes. The finding that healthcare access ranks as the third most influential feature (importance = 0.15) supports the argument that climate impacts can be mitigated, to some extent, through institutional capacity and governance quality [13]. This aligns with prior empirical work demonstrating that countries with stronger universal health coverage (UHC) indices experience up to 40% lower mortality from climate-related disasters [21]. As such, the current analysis highlights that planetary health resilience is not solely a function of environmental thresholds but also of political and social adaptability.

Importantly, this study quantifies a growing but often overlooked domain: the mental health implications of climate change. The observed 32% increase in climate-attributable mental health DALYs at 2.5°C warming underscores the psychological dimension of the climate crisis. The evidence supports a bidirectional relationship—extreme heat and environmental disruption trigger anxiety, depression, and post-traumatic stress, while deteriorating mental health weakens adaptive capacity, creating feedback loops that reinforce vulnerability. This complements emerging research showing that suicide rates and psychological distress rise significantly during prolonged heatwaves and climate-related disasters [23]. Thus, integrating mental health into climate adaptation planning should be seen as a public health priority rather than a peripheral issue.

From a systems perspective, the results suggest that crossing planetary boundaries introduces nonlinearities and feedbacks that traditional global burden models have struggled to incorporate. The transition from incremental to exponential health impacts mirrors other ecological thresholds, such as those observed in coral bleaching or polar ice loss, implying that the health system may exhibit similar “state shifts” under sustained environmental stress [24,25]. These findings advance the concept of “planetary health feedback loops,” where ecological degradation and human health decline reinforce each other [4,25]. As such, this study contributes to an emerging body of literature advocating for a unified planetary health framework that integrates environmental limits with population-level health metrics.

Finally, the study’s projections have strong implications for policy. Limiting global warming below 2.0°C is not only an environmental imperative but a public health necessity that could prevent millions of premature deaths and DALY losses. Policy efforts should therefore prioritize co-beneficial interventions that simultaneously reduce emissions and improve health—such as transitioning to clean energy, promoting active transport, and strengthening primary health systems [26,27]. Equally crucial is the need for health system adaptation: enhancing surveillance for climate-sensitive diseases, embedding AI-based early warning systems, and expanding health financing in vulnerable regions. Without such actions, the transgression of planetary boundaries may translate into an irreversible global health crisis [28,29].

### 4.1 Limitations

This study has several limitations. Although it integrates multiple global datasets, variations in data quality—especially in LMICs—may lead to underestimation of disease burdens. The AI models, while capturing complex non-linear relationships, remain data-driven and dependent on current assumptions, limiting their ability to account for future adaptation, behavioral change, or technological advances. The analysis focused primarily on temperature as a marker of planetary boundary transgression, without fully modeling other ecological stressors such as biodiversity loss, ocean acidification, or soil degradation that interact with human health. Furthermore, while SHAP and causal inference improve interpretability, they provide probabilistic rather than deterministic causal attributions. Although structural causal models and DAGs were employed to organize and constrain the analysis, results should interpret as observational and simulation-based and should be understood in the context of a mechanistically informed, model-based framework rather than a randomized or quasi-experimental design. Lastly, projections rely on current SSP/RCP scenarios and may shift with evolving global mitigation and socioeconomic trends.

## 5. Conclusion

Crossing the planetary boundary for climate change represents not just an environmental milestone but a fundamental turning point in global health. This study demonstrates that beyond 1.5°C of warming, health impacts accelerate exponentially, threatening to overwhelm health systems and reverse decades of development gains. The disproportionate burden on vulnerable regions and populations highlights the intersection of environmental and social inequities that define the modern planetary health crisis. Maintaining global warming below 2°C could avert millions of premature deaths and disability years, yielding one of the most profound public health dividends in history. This will require simultaneous action on two fronts: rapid decarbonization to restore Earth system stability and strategic investment in climate-resilient, equitable health systems. Artificial intelligence and data-driven modeling can play a transformative role in guiding this transition by improving foresight, prioritizing interventions, and strengthening accountability. Ultimately, planetary stability and human health are interdependent systems—preserving one safeguards the other. The challenge before us is not only scientific but ethical: to ensure that the boundaries of our planet remain compatible with the boundaries of human survival.

## Abbreviations

AI: Artificial Intelligence
AUC: Area Under the Receiver Operating Characteristic Curve
CMIP6: Coupled Model Intercomparison Project Phase 6
CNN-LSTM: Convolutional Neural Network-Long Short-Term Memory
COPD: Chronic Obstructive Pulmonary Disease
CVD: Cardiovascular Disease
DALY: Disability-Adjusted Life Year (one DALY = one year of healthy life lost due to premature death or disability)
DAG: Directed Acyclic Graph
ERA5: Fifth Generation ECMWF Atmospheric Reanalysis
F1: F1-Score
GBD: Global Burden of Disease
GMST: Global Mean Surface Temperature
GBV: Gender-Based Violence
HI: Heat Index
IHME: Institute for Health Metrics and Evaluation
IPCC: Intergovernmental Panel on Climate Change
IPW: Inverse Probability Weighting
LMIC: Low- and Middle-Income Country
MAE: Mean Absolute Error
ML: Machine Learning
PM2.5: Fine Particulate Matter
ppm: Parts Per Million
PTSD: Post-Traumatic Stress Disorder
R^2^: Coefficient of Determination
RCP: Representative Concentration Pathway
RF: Random Forest
RMSE: Root Mean Square Error
SDI: Socio-Demographic Index
SHAP: SHapley Additive exPlanations
SSP: Shared Socioeconomic Pathway
UHC: Universal Health Coverage
UI: Uncertainty Interval
UNDRIP: United Nations Declaration on the Rights of Indigenous Peoples
VBD: Vector-Borne Disease
WASH: Water, Sanitation and Hygiene
WHO: World Health Organization
XGBoost: Extreme Gradient Boosting
YLD: Years Lived with Disability
YLL: Years of Life Lost
