# Supplementary material for: Beyond the 1.5°C planetary boundary: AI-forecast of Global Disease Burden and Health inequities
Source: PLoS One. 2026 Aug 10;21(8):e0354159. doi: 10.1371/journal.pone.0354159 (PMC13456168; doi:10.1371/journal.pone.0354159)
Supplement: Supplementary file 1 [file pone.0354159.s001.docx]

**Iskra Alexandra Nola**

**Academic Editor**

**PLOS One**

**Re: Revised Manuscript — “Beyond the 1.5°C Planetary Boundary: AI-forecast of Global Disease Burden and Health Inequities” (PONE-D-26-09733)**

Dear Editor,

We are grateful to the Editors and both Reviewers for their constructive and thorough critique. We have carefully addressed each point raised. All changes are highlighted in yellow in the revised manuscript. A point-by-point response follows below.

# **Response to Reviewer #1**

**R1 Comment 1***: “DAGs mentioned but not shown or described clearly.”*

**Response:** We have substantially expanded Section 2.2.2.2 (Causal Inference Modeling) to describe the DAG structure in detail.

**R1 Comment 2***: “No identification strategy (e.g., confounding control).”*

**Response:** In the same expanded passage (Section 2.2.2.2), we now explicitly state that confounding was controlled by conditioning on SDI, healthcare access index, population density, and geographic region as adjustment variables.

**R1 Comment 3***: “Statements imply causality, but models are largely predictive.”*

**Response:** We added explicit caveats in Section 2.2.2.2 and in the Limitations.

**R1 Comment 4***: “No confidence intervals for projections; no uncertainty bounds from models.”*

**Response:** We have added 95% uncertainty intervals (UI) to the opening of the Results section (Section 3, first paragraph) and to all key projections.

**R1 Comment 5***: “Figure 4 (SHAP plot) is not fully interpreted.”*

**Response:** We have added a detailed interpretive paragraph immediately following the Figure 4 feature-list text in Section 3.8 (AI Framework Insights and SHAP Feature Importance).

# **Response to Reviewer #2**

**R2 Comment 1***: “The authors need to do a lot more justification for the approaches used. This should be in the Methods section.”*

**Response:** The methodological justification has been strengthened via the expanded DAG/causal inference description in Section 2.2.2.2.

**R2 Comment 2***: “There are a very large number of abbreviations used and not explained. The most irritating here is ‘DALY’ which is never explained. The authors need to put in a glossary of abbreviations.”*

**Response:** We have added a Glossary of Abbreviations has been added as a new section immediately after the Conclusion section.

**R2 Comment 3***: “Lines 460–462: Why not refer to the 2.5 data as well (isn’t this worse)?”*

**Response:** We agree entirely. The Discussion opening sentence revised to the 1.5°C, 2.0°C, and 2.5°C global warming thresholds.

**R2 Comment 4***: “The authors use the term ‘aboriginal’. This in Australia and maybe elsewhere is derogatory to some. The term needs to be defined or changed to, say ‘First Nations’ people.”*

**Response:** We thank the reviewer for this important cultural sensitivity note. Upon review, the term “aboriginal” does not appear in the manuscript; the original text uses “Indigenous populations” and “Indigenous peoples.” However, we have taken the spirit of the comment seriously and made two improvements: (1) the label has been standardised throughout to “Indigenous Peoples” (capitalised, per UNDRIP convention) and that “Indigenous Peoples” is used in accordance with the United Nations Declaration on the Rights of Indigenous Peoples (UNDRIP) and preferred international usage.

We believe the revised manuscript is substantially stronger as a result of the reviewers’ feedback. All changes are clearly marked in yellow highlight throughout the document. We hope the revisions meet the standards required for publication in PLOS ONE and look forward to your decision.
