# Supplementary material for: Beyond the 1.5°C planetary boundary: AI-forecast of Global Disease Burden and Health inequities
Source: PLoS One. 2026 Aug 10;21(8):e0354159. doi: 10.1371/journal.pone.0354159 (PMC13456168; doi:10.1371/journal.pone.0354159)
Supplement: Peer review history

# Peer review: Decision Letter 0

**DOI:** 10.1371/journal.pone.0354159.r001

**Authors:** Iskra Alexandra Nola

5 May 2026

PONE-D-26-09733

Beyond the 1.5°C Planetary Boundary: AI-forecast of Global Disease Burden and Health Inequities

PLOS One

Dear Dr. Rahman,

Thank you for submitting your manuscript to PLOS ONE. After careful consideration, we feel that it has merit but does not fully meet PLOS ONE’s publication criteria as it currently stands. Therefore, we invite you to submit a revised version of the manuscript that addresses the points raised during the review process.

I would especially like you to read the reviews thoroughly and improve your paper accordingly. Especially in the methods section, to meet the publication standards.

Your paper is very interesting, not only in terms of the valuable topic but also in terms of the chosen methodology. Therefore, it needs to be revised based on the reviewers' comments. In addition, the methodology and results need to be harmonized with all explanations.

Please submit your revised manuscript by the Jun 19 2026 11:59PM. If you will need more time than this to complete your revisions, please reply to this message or contact the journal office at plosone@plos.org. When you're ready to submit your revision, log on to https://www.editorialmanager.com/pone/ and select the 'Submissions Needing Revision' folder to locate your manuscript file.

Please include the following items when submitting your revised manuscript:

- A letter that responds to each point raised by the academic editor and reviewer(s). You should upload this letter as a separate file labeled 'Response to Reviewers'.
- A marked-up copy of your manuscript that highlights changes made to the original version. You should upload this as a separate file labeled 'Revised Manuscript with Track Changes'.
- An unmarked version of your revised paper without tracked changes. You should upload this as a separate file labeled 'Manuscript'.

If you would like to make changes to your financial disclosure, please include your updated statement in your cover letter. Guidelines for resubmitting your figure files are available below the reviewer comments at the end of this letter.

If applicable, we recommend that you deposit your laboratory protocols in protocols.io to enhance the reproducibility of your results. Protocols.io assigns your protocol its own identifier (DOI) so that it can be cited independently in the future. For instructions see: https://journals.plos.org/plosone/s/submission-guidelines#loc-laboratory-protocols. Additionally, PLOS ONE offers an option for publishing peer-reviewed Lab Protocol articles, which describe protocols hosted on protocols.io. Read more information on sharing protocols at https://plos.org/protocols?utm_medium=editorial-email&utm_source=authorletters&utm_campaign=protocols.

As the corresponding author, your ORCID iD is verified in the submission system and will appear in the published article. PLOS supports the use of ORCID, and we encourage all coauthors to register for an ORCID iD and use it as well. Please encourage your coauthors to verify their ORCID iD within the submission system before final acceptance, as unverified ORCID iDs will not appear in the published article. *Only*  the individual author can complete the verification step; PLOS staff *cannot*  verify ORCID iDs on behalf of authors.

We look forward to receiving your revised manuscript.

Kind regards,

Iskra Alexandra Nola

Academic Editor

PLOS One

Journal Requirements:

When submitting your revision, we need you to address these additional requirements.

1. Please ensure that your manuscript meets PLOS ONE's style requirements, including those for file naming. The PLOS ONE style templates can be found at

https://journals.plos.org/plosone/s/file?id=wjVg/PLOSOne_formatting_sample_main_body.pdf and

https://journals.plos.org/plosone/s/file?id=ba62/PLOSOne_formatting_sample_title_authors_affiliations.pdf

2. We note that your Data Availability Statement is currently as follows: "Source of Data can be found within manuscript."

Please confirm at this time whether or not your submission contains all raw data required to replicate the results of your study. Authors must share the “minimal data set” for their submission. PLOS defines the minimal data set to consist of the data required to replicate all study findings reported in the article, as well as related metadata and methods (https://journals.plos.org/plosone/s/data-availability#loc-minimal-data-set-definition).

For example, authors should submit the following data:

- The values behind the means, standard deviations and other measures reported;

- The values used to build graphs;

- The points extracted from images for analysis.

Authors do not need to submit their entire data set if only a portion of the data was used in the reported study.

If your submission does not contain these data, please either upload them as Supporting Information files or deposit them to a stable, public repository and provide us with the relevant URLs, DOIs, or accession numbers. For a list of recommended repositories, please see https://journals.plos.org/plosone/s/recommended-repositories.

If there are ethical or legal restrictions on sharing a de-identified data set, please explain them in detail (e.g., data contain potentially sensitive information, data are owned by a third-party organization, etc.) and who has imposed them (e.g., an ethics committee). Please also provide contact information for a data access committee, ethics committee, or other institutional body to which data requests may be sent. If data are owned by a third party, please indicate how others may request data access.

3. If the reviewer comments include a recommendation to cite specific previously published works, please review and evaluate these publications to determine whether they are relevant and should be cited. There is no requirement to cite these works unless the editor has indicated otherwise.

[Note: HTML markup is below. Please do not edit.]

Reviewers' comments:

Reviewer's Responses to Questions

**Comments to the Author**

1. Is the manuscript technically sound, and do the data support the conclusions?

The manuscript must describe a technically sound piece of scientific research with data that supports the conclusions. Experiments must have been conducted rigorously, with appropriate controls, replication, and sample sizes. The conclusions must be drawn appropriately based on the data presented.

Reviewer #1: Yes

Reviewer #2: Yes

**********

2. Has the statistical analysis been performed appropriately and rigorously?

Reviewer #1: Yes

Reviewer #2: Yes

**********

3. Have the authors made all data underlying the findings in their manuscript fully available?

The PLOS Data policy requires authors to make all data underlying the findings described in their manuscript fully available without restriction, with rare exception (please refer to the Data Availability Statement in the manuscript PDF file). The data should be provided as part of the manuscript or its supporting information, or deposited to a public repository. For example, in addition to summary statistics, the data points behind means, medians and variance measures should be available. If there are restrictions on publicly sharing data—e.g. participant privacy or use of data from a third party—those must be specified.

Reviewer #1: Yes

Reviewer #2: Yes

**********

4. Is the manuscript presented in an intelligible fashion and written in standard English?

PLOS ONE does not copyedit accepted manuscripts, so the language in submitted articles must be clear, correct, and unambiguous. Any typographical or grammatical errors should be corrected at revision, so please note any specific errors here.

Reviewer #1: Yes

Reviewer #2: Yes

**********

5. Review Comments to the Author

Please use the space provided to explain your answers to the questions above. You may also include additional comments for the author, including concerns about dual publication, research ethics, or publication ethics. (Please upload your review as an attachment if it exceeds 20,000 characters)

Reviewer #1: Overall Assessment

This manuscript addresses a highly relevant global health issue using an innovative AI-driven framework. It demonstrates strong novelty and policy relevance but requires major revisions, particularly in the methods section, to meet publication standards.

Specific Section Comments

Methods (Critical Revision Required)

1. DAGs mentioned but not shown or described clearly

2. No identification strategy (e.g., confounding control)

3. Statements imply causality, but models are largely predictive

Results

Results are well presented but lack uncertainty intervals.

2. Missing Statistical Uncertainty

1. No confidence intervals for projections

2. No uncertainty bounds from models

3. Figures Not Fully Interpreted

• Example: SHAP plot (page 17)

Reviewer #2: This submission seems to be a rational and statistical analysis of the potential for a 1.5, 2.0 and 2.5 degree rise in Global temperature. The authors use Ai-genrated approaches with which I am not familiar but these seem to be applicable. However the authors need to do a lot more justification for the approaches used. This should be in the Methods section.

There are a very large number of abbreviations used and not explained. The most irritating here is "DALY" which is never explained. However there are many more. The authors need to put in a glossary of abbreviations

The submission is very detailed and complicated. I have tried understand the detailed work and its intersections but I have not had time to check (cross-reference all the long explanations. This need to be done by an editor..

Minor points

Lines 460-461. This study provides an integrated, data-driven analysis of how surpassing planetary climate boundaries—

462 particularly the 1.5°C and 2.0°C global warming thresholds—exponentially accelerates the global burden

Why not refer to the 2.5 data as well (isn't this worse?

The authors use the term "aboriginal". This in Australia and maybe elsewhere is derogatory to some. The term needs to be defined or changed to, say "First Nations" people.

**********

6. PLOS authors have the option to publish the peer review history of their article (what does this mean?). If published, this will include your full peer review and any attached files.

If you choose “no”, your identity will remain anonymous but your review may still be made public.

**Do you want your identity to be public for this peer review?**  For information about this choice, including consent withdrawal, please see our Privacy Policy.

Reviewer #1: No

Reviewer #2: **Yes:**  Anthony Larkum

**********

[NOTE: If reviewer comments were submitted as an attachment file, they will be attached to this email and accessible via the submission site. Please log into your account, locate the manuscript record, and check for the action link "View Attachments". If this link does not appear, there are no attachment files.]

To ensure your figures meet our technical requirements, please review our figure guidelines: https://journals.plos.org/plosone/s/figures

You may also use PLOS’s free figure tool, NAAS, to help you prepare publication quality figures: https://journals.plos.org/plosone/s/figures#loc-tools-for-figure-preparation.

NAAS will assess whether your figures meet our technical requirements by comparing each figure against our figure specifications.

# Author comment: Author response to Decision Letter 1

**DOI:** 10.1371/journal.pone.0354159.r002

9 May 2026

Response to Reviewer #1

R1 Comment 1: “DAGs mentioned but not shown or described clearly.”

Response: We have substantially expanded Section 2.2.2.2 (Causal Inference Modeling) to describe the DAG structure in detail.

R1 Comment 2: “No identification strategy (e.g., confounding control).”

Response: In the same expanded passage (Section 2.2.2.2), we now explicitly state that confounding was controlled by conditioning on SDI, healthcare access index, population density, and geographic region as adjustment variables.

R1 Comment 3: “Statements imply causality, but models are largely predictive.”

Response: We added explicit caveats in Section 2.2.2.2 and in the Limitations.

R1 Comment 4: “No confidence intervals for projections; no uncertainty bounds from models.”

Response: We have added 95% uncertainty intervals (UI) to the opening of the Results section (Section 3, first paragraph) and to all key projections.

R1 Comment 5: “Figure 4 (SHAP plot) is not fully interpreted.”

Response: We have added a detailed interpretive paragraph immediately following the Figure 4 feature-list text in Section 3.8 (AI Framework Insights and SHAP Feature Importance).

Response to Reviewer #2

R2 Comment 1: “The authors need to do a lot more justification for the approaches used. This should be in the Methods section.”

Response: The methodological justification has been strengthened via the expanded DAG/causal inference description in Section 2.2.2.2.

R2 Comment 2: “There are a very large number of abbreviations used and not explained. The most irritating here is ‘DALY’ which is never explained. The authors need to put in a glossary of abbreviations.”

Response: We have added a Glossary of Abbreviations has been added as a new section immediately after the Conclusion section.

R2 Comment 3: “Lines 460–462: Why not refer to the 2.5 data as well (isn’t this worse)?”

Response: We agree entirely. The Discussion opening sentence revised to the 1.5°C, 2.0°C, and 2.5°C global warming thresholds.

R2 Comment 4: “The authors use the term ‘aboriginal’. This in Australia and maybe elsewhere is derogatory to some. The term needs to be defined or changed to, say ‘First Nations’ people.”

Response: We thank the reviewer for this important cultural sensitivity note. Upon review, the term “aboriginal” does not appear in the manuscript; the original text uses “Indigenous populations” and “Indigenous peoples.” However, we have taken the spirit of the comment seriously and made two improvements: (1) the label has been standardised throughout to “Indigenous Peoples” (capitalised, per UNDRIP convention) and that “Indigenous Peoples” is used in accordance with the United Nations Declaration on the Rights of Indigenous Peoples (UNDRIP) and preferred international usage.

Attachment Submitted filename: Response to reviewers.docx

# Editor report: Decision Letter 1

**DOI:** 10.1371/journal.pone.0354159.r003

**Authors:** Iskra Alexandra Nola

6 Jul 2026

Beyond the 1.5°C Planetary Boundary: AI-forecast of Global Disease Burden and Health Inequities

PONE-D-26-09733R1

Dear Dr. Rahman,

We’re pleased to inform you that your manuscript has been judged scientifically suitable for publication and will be formally accepted for publication once it meets all outstanding technical requirements.

Within one week, you’ll receive an e-mail detailing the required amendments. When these have been addressed, you’ll receive a formal acceptance letter and your manuscript will be scheduled for publication.

An invoice will be generated when your article is formally accepted. Please note, if your institution has a publishing partnership with PLOS and your article meets the relevant criteria, all or part of your publication costs will be covered. Please make sure your user information is up-to-date by logging into Editorial Manager at Editorial Manager® and clicking the ‘Update My Information' link at the top of the page. For questions related to billing, please contact billing support.

If your institution or institutions have a press office, please notify them about your upcoming paper to help maximize its impact. If they’ll be preparing press materials, please inform our press team as soon as possible -- no later than 48 hours after receiving the formal acceptance. Your manuscript will remain under strict press embargo until 2 pm Eastern Time on the date of publication. For more information, please contact onepress@plos.org.

Kind regards,

Iskra Alexandra Nola

Academic Editor

PLOS One

Additional Editor Comments (optional):

In the revised version, you added text (lines 422 to 438) describing the SHAP plot, but I cannot find the plot itself. Could you please relate this text to an adequate picture?

Reviewers' comments:

# Editor report: Acceptance letter

**DOI:** 10.1371/journal.pone.0354159.r004

**Authors:** Iskra Alexandra Nola

PONE-D-26-09733R1

PLOS One

Dear Dr. Rahman,

I'm pleased to inform you that your manuscript has been deemed suitable for publication in PLOS One. Congratulations! Your manuscript is now being handed over to our production team.

At this stage, our production department will prepare your paper for publication. This includes ensuring the following:

* All references, tables, and figures are properly cited

* All relevant supporting information is included in the manuscript submission,

* There are no issues that prevent the paper from being properly typeset

You will receive further instructions from the production team, including instructions on how to review your proof when it is ready. Please keep in mind that we are working through a large volume of accepted articles, so please give us a few days to review your paper and let you know the next and final steps.

Lastly, if your institution or institutions have a press office, please let them know about your upcoming paper now to help maximize its impact. If they'll be preparing press materials, please inform our press team within the next 48 hours. Your manuscript will remain under strict press embargo until 2 pm Eastern Time on the date of publication. For more information, please contact onepress@plos.org.

You will receive an invoice from PLOS for your publication fee after your manuscript has reached the completed accept phase. If you receive an email requesting payment before acceptance or for any other service, this may be a phishing scheme. Learn how to identify phishing emails and protect your accounts at https://explore.plos.org/phishing.

If we can help with anything else, please email us at customercare@plos.org.

Thank you for submitting your work to PLOS ONE and supporting open access.

Kind regards,

PLOS ONE Editorial Office Staff

on behalf of

Dr. Iskra Alexandra Nola

Academic Editor

PLOS One
